# Intrathecal/Intraventricular Colistin for Antibiotic-Resistant Bacterial CNS Infections in Pediatric Population: A Systematic Review

**DOI:** 10.3390/tropicalmed7030041

**Published:** 2022-03-03

**Authors:** Ibrahim Alnaami, Zubaidah Alahmari

**Affiliations:** 1 Department of Surgery, Division of Neurosurgery, King Khalid University, Abha 61421, Saudi Arabia; 2 Department of Pediatric Neurosurgery, Abha Maternity and Children Hospital, Abha 61421, Saudi Arabia; 3 Department of Neurosurgery, Aseer Central Hospital, Abha 61421, Saudi Arabia; 4 Department of Pediatric Neurosurgery, Khamis Mushait Maternity and Children Hospital, Khamis Mushait 61961, Saudi Arabia; zualahmari@moh.gov.sa

**Keywords:** CNS infection, children, colistin, *Acinetobacter baumannii*, intrathecal, intraventricular

## Abstract

Central nervous system (CNS) infections constitute a life-threatening condition, especially in children. Treatment limitations exist for drug-resistant CNS bacterial infections. Inadequate CNS penetration and intravenous (IV) antibiotic treatment failure represent a major clinical challenge. However, patients with antibiotic-resistant bacterial CNS infections may benefit from intrathecal (IT) or intraventricular (IVT) colistin. The authors aimed to assess the safety and effectiveness of IT/IVT colistin therapy in the pediatric population, with or without other antibiotics, for the treatment of antibiotic-resistant CNS infections. A comprehensive literature search was conducted using the electronic databases of PubMed, Ovid, and Embase for relevant articles using the following terms: “Colistin”, “CNS infection”, and “Outcome”, as well as their combinations. The retrieved articles were filtered by age (Child), language (English), route of administration (IT/IVT), and species (Humans). The present systematic review comprised 20 articles that included 31 children (19; 61.2% were boys) with multidrug-resistant CNS infection. Their ages ranged from less than one month to 18 years (median: 9 months). *Acinetobacter baumannii* was the main causative organism in 22 patients (70.9%), and infection occurred mainly after neurosurgical interventions (83.8%). An external ventricular drain was inserted to administer colistin into the ventricular system in 29 cases (93.5%). The median duration for colistin therapy was 18 days. Twenty-three patients (74%) recovered, while five patients (16%) had residual disability, and three patients (10%) died. The authors concluded that IT/IVT colistin therapy is safe and effective as either the primary or adjunct treatment for antibiotic-resistant cases with CNS infection.

## 1. Introduction

Infections of the central nervous system (CNS) may develop in patients with traumatic brain injury after external ventricular drainage insertion and, broadly, after any neurosurgery procedure. Various bacteria are responsible for these infections, especially *Staphylococcus epidermidis*, but Gram-negative bacteria are responsible for almost 15% of CNS infections. Many patients with CNS infections develop systemic and neurologic complications, with potential sequelae in the survivors and substantial mortality. These infections require prompt diagnosis and management, which depend mainly on the local epidemiology and patterns of antibiotic resistance of common pathogens [1,2].

It has been shown that the extensive use of antibiotics after neurosurgical CNS infections might alter their epidemiology. External ventricular drainage is one of the common procedures performed in neurosurgery associated with a variable infection rate ranging from 2% to 33% [3].

Due to the increasing emergence of multidrug-resistant bacteria (MDR) (e.g., *Acinetobacter*, *A. baumannii*, *Pseudomonas* *aeruginosa*, and *Klebsiella pneumoniae*) [4,5], treatment of CNS infections has become increasingly difficult. Several bacteria have become susceptible only to colistin and polymyxin B. Colistin is a complex mixture of polymyxins, mainly polymyxin E1 and polymyxin E2. It was the first antibiotic with notable in vitro activity against *P. aeruginosa* [1].

Colistin began to be used in the treatment of multidrug-resistant *A. baumannii* infections [6]. However, since there is a limited penetration of intravenous colistin to the cerebrospinal fluid, IT or IVT treatment is commonly applied in CNS infections [7].

This study aimed to assess the effectiveness of IT/IVT colistin therapy, with or without other antibiotics, for the treatment of antibiotic-resistant CNS infections in the pediatric population, as the existing evidence in the literature is mostly about adult patients, with little emphasis on pediatric patients apart from scattered case reports and case series [6].

## 2. Materials and Methods

**Figure 1:**
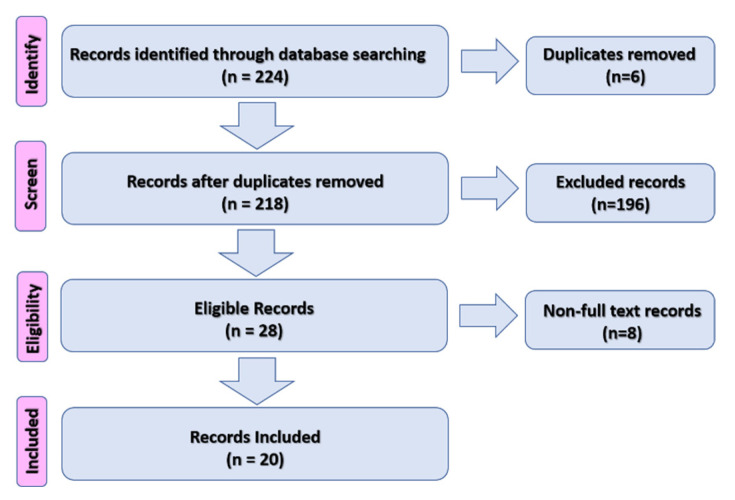
Flow chart of the study procedures.

We conducted a comprehensive literature search using the electronic databases of PubMed, Ovid, and Embase for relevant articles during the period 2000–2021. The following terms were used: “Colistin”, “CNS infection”, and “Outcome”, as well as their combinations. A total of 244 articles was retrieved. After the removal of duplicates (six articles), the articles were filtered by age (Child), language (English), and species (Humans); 28 articles were eligible. However, eight articles were also removed because of the unavailability of the full text. Finally, this study included 20 articles with 31 cases (Figure 1). The PRISMA 2020 checklist is attached in the Supplementary Materials.

## 3. Results

The present systematic review comprised 20 articles that included 31 children (16; 61.2% were boys) with CNS infections. Table 1 shows the summary of the main findings of included studies.

Their ages ranged from less than one month to 18 years (median: 9 months). In all cases, IT/IVT colistin was administered.

**Figure 2:**
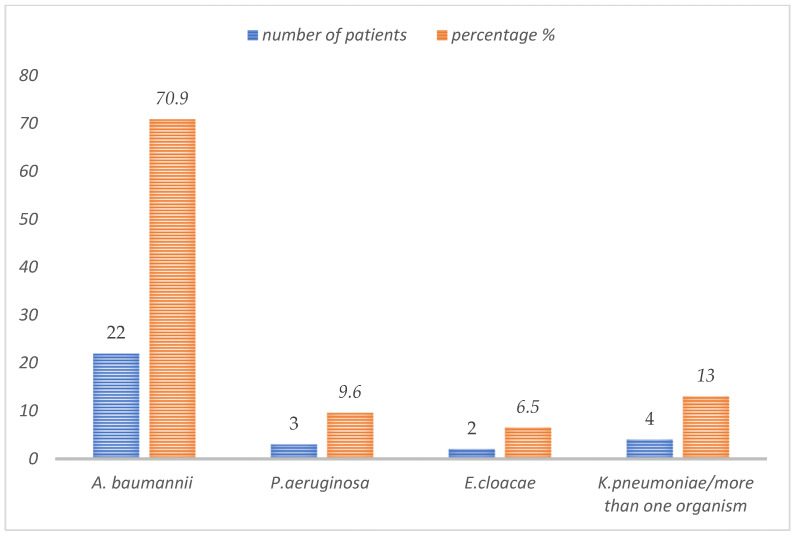
Causative organisms of CNS infections in pediatric patients treated with IV/IT colistin.

*Acinetobacter baumannii* was the main causative organism in 22 cases (70.9%), while *P. aeruginosa* was the causative organism in three cases (9.6%), *Enterobacter cloacae* was the causative organisms in two cases (≈6.5%), and more than one organism was seen in three patients (9.6%). *K. pneumoniae* was the causative organism in four cases (≈13%); however, three of them were associated with another organism (Figure 2).

The route of infection in the review cases was mainly after neurosurgical interventions (26 cases, 83.8%). An external ventricular drain was inserted in 29 cases (93.5%). The median duration for colistin therapy was 18 days. Twenty-three patients (74%) recovered, while five patients (16%) had residual disability, and three patients (10%) died.

## 4. Discussion

CNS infections are associated with high morbidity and the case fatality rate, in addition to a significant cost to the healthcare system.

Despite colistin being introduced in clinical practice in 1947, it was not until recent years that concerns were raised regarding the low level of colistin concentration in the cerebrospinal fluid, leading to a concern for poor penetration of the blood–brain barrier, which has led to the possibility of IT/IVT administration to achieve direct eradication of the causative organism [6]. It was also reported that only 5–25% of IV colistin crosses into the CSF [18]. Even in patients with meningitis, with a disrupted blood–brain barrier, this rate does not exceed 25%. On the other hand, several studies proved the efficacy of IT and IVT colistin administration [14,26]. However, the first report of intrathecal administration of colistin in a pediatric patient was back in 1990 [8].

In recent years, *A. baumannii* has appeared as an important pathogen, especially in hospital-acquired infections [27]. The risk factors for infection are neurosurgical procedures, CSF leak, a prolonged use of intravascular devices, prior treatment with broad-spectrum antibiotics, prior colonization with *Acinetobacter*, prolonged mechanical ventilation, and ICU stay, as well as intracranial hemorrhage [6].

It was reported that the incidence of CNS infections is associated with ventricular devices, with more than 50% of the episodes occurring within the first month of implant placement [17]. Therefore, it is generally advised in cases of CSF shunt infections that all components of infected shunts or external drainage devices be removed and appropriate antimicrobial therapy be administered in combinations [28].

In the USA, there are no FDA-approved antimicrobial agents for intraventricular use [25]. Intraventricular colistin is being used off-label to sterilize the cerebrospinal fluid. Moreover, the dosages for antimicrobials have been empiric, with dose adjustments and dosing intervals mainly based on pharmacokinetic calculations to achieve adequate CNS concentrations [28].

Polymyxins are increasingly used for the treatment of CNS infections caused by multidrug-resistant Gram-negative bacteria. Four polymyxins exhibit rapid bacterial killing against most Gram-negative microorganisms by disturbing the bacterial cell membrane and can block the biologic effects of endotoxins [26]. However, intravenously administered polymyxins do not achieve high concentrations in the CNS. Therefore, IVT and ITH have been recommended in patients with CNS device infections that are difficult to eradicate or who cannot undergo the surgical removal of infected devices [7].

Many studies addressed the safety and effectiveness of IT/IVT colistin administration in adults, and some of these studies included pediatric patients. However, no study was encountered during our search that focused on the pediatric population, apart from case reports and case series or being involved with adult patients in some review [6].

The authors noticed a drop in the number of published cases of IT/IVT colistin in the pediatric population over the last five years in comparison to the years before. This may reflect that clinicians are widely using it, being convinced of its safety from the existing literature, or it may reflect the flip side of the coin, where it is not widely accepted in practice.

The toxicity from intrathecal or intraventricular therapy with colistin is a major challenge, especially with respect to aseptic or chemical meningitis, which is very hard to differentiate clinically from bacterial meningitis. However, the CSF parameters are the only key in differentiating the two [6]. Despite that, the three deaths that were encountered in our review were not related directly to the intrathecal toxicity, as one child, for instance, died from consequences of hydrocephalus, and the team with the family reached the conclusion of withdrawing care after the EVD was inadvertently dislodged [25], and another child died from the consequences of sepsis [15].

A recent study addressed the use of colistin in neonates, which is one of the largest series of its kind, and the authors found that six out of seven patients who received IT/IVT colistin survived, where five of them received it in combination with IV colistin, and the one that died received only IT/IVY colistin [29]. The study went on further to report the neurodevelopmental outcome. The authors reported three patients who had a seizure disorder on follow-up, and the only patient with moderate disability had both hearing and visual impairments [29].

The current review is the first of its kind to address the safety and effectiveness of IT/IVT colistin in the pediatric population. Despite the mortality and disability that were reported, it is difficult to determine whether this is a result of the primary pathology or adverse effects of colistin [10]. The authors believe that IT/IVT as a last resort of the management of serious CNS infections should be pursued without delay. However, caution throughout the treatment period should be implemented by all treating teams, which should include pediatric neurosurgeons, infectious disease specialists, neurologists, clinical pharmacologists, etc.

There was no consistency regarding the dosage of IT/IVT used in these studies. However, a dose of 0.16–0.24 mg/kg every 24 h was most commonly used [6,15]. Some studies used up to 10 mg every 24 h, which appeared to be a high dose in, for instance, a three-year-old child; despite that, it is hard to comment on the appropriateness of the dose with the lack of data on the weight of children [13].

## 5. Limitations of the Review

The small number of patients was one of the challenges encountered. However, based on the search flowchart, we included all studies that met the criteria, and to the best of our knowledge, this review has the biggest number of pediatric patients who received IT/IVT in the literature. The other challenge is the inconsistency in the reporting of the time of initiation of IV/IVT, whether it was immediately after diagnosis or after failure of intravenous treatment; in the case of the latter, there is also inconsistency regarding the wait time for the intravenous medication before declaring its failure and, consequently, starting IV/IVT colistin.

The authors recommend further studies and suggest that a well-structured randomized control trial may be able address this concern.

## 6. Conclusions

IT/IVT colistin therapy is a safe and effective last resort of management as either the primary or adjunct treatment for antibiotic-resistant cases with CNS infections in the pediatric population.

## Figures and Tables

**Table 1:** Summary of the main findings of the included studies.

Reference	Age	Gender	Route of Infection	EVD	CSF Culture	Colistin (Duration)	Other Antibiotics	Outcome
Kaplan & Patrick, 1990 [8]	4 years	NR	CSF leak after trauma	Yes	*A. baumannii*	IV, IVT, and IT (20 days)	--	Recovered
Fernandez-Viladrich et al., 1999 [9]	16 years	Male	After neurosurgical intervention	Yes	*A. baumannii*	IVT and IT (20 days)	Meropenem, Tobramycin, Sulbactam	Severe disability
Ng et al., 2006 [10]	4 years	Male	After neurosurgical intervention	No	*A. baumannii*	IV, IVT, and IT (24 days)	Amikacin	Severe disability
Yagmur & Esen, 2006 [11]	16 years	Male	After neurosurgical intervention	Yes	*P. aeruginosa*	IVT and IT (21 days)	IV amikacin	Recovered
Dalgic et al., 2009 [12]	2 months	Female	After neurosurgical intervention	Yes	*A. baumannii*	IV, IVT, and IT (27 days)	--	Recovered
Dalgic et al., 2009 [12]	2 months	Female	After neurosurgical intervention	Yes	*K. pneumoniae*	IV, IVT, and IT (14 days)	Ciprofloxacin	Recovered
Özdemir et al., 2010 [13]	3 years	Female	After neurosurgical intervention	Yes	*A. baumannii*	IV, IVT, and IT (35 days)	Meropenem, amikacin, ampicillin	Recovered
Cascio et al., 2010 [14]	5 years	Male	After neurosurgical intervention	Yes	*Enterobacter cloacae*	IV, IVT, and IT (14 days)	Teicoplanin, Rifampin, cefazidime	Moderate disability
Saleem et al., 2011 [15]	5 months	Male	After neurosurgical intervention	Yes	*A. baumannii*	IV, IVT, and IT (24 days)	--	Died
Saleem et al., 2011 [15]	9 months	Male	After neurosurgical intervention	No	*A. baumannii*	IV, IVT, and IT (6 days)	--	Recovered
Saleem et al., 2011 [15]	3 months	Female	After neurosurgical intervention	Yes	*A. baumannii*	IV, IVT, and IT (11 days)	--	Recovered
Saleem et al., 2011 [15]	9 years	Female	After neurosurgical intervention	Yes	*A. baumannii*	IV, IVT, and IT (21 days)	--	Recovered
Wang et al., 2012 [16]	15 years	Male	After neurosurgical intervention	Yes	*A. baumannii*	IV, IVT, and IT (24 days)	Meropenem	Recovered
Karaiskos et al., 2013 [6]	18 years	Female	After neurosurgical intervention	Yes	*A. baumannii*	IV, IVT, and IT (17 days)	Carbapenem, Sulbactam.	Recovered
Bargiacchi et al., 2014 [17]	18 years	Male	After neurosurgical intervention	Yes	*P. aeruginosa*	IV, IVT, and IT (18 days)	Ciprofloxacin	Recovered
Tekgündüz et al., 2015 [18]	<1 month	Male	After neurosurgical intervention	Yes	*A. baumannii*	IV, IVT, and IT (9 days)	Vancomycin	Recovered
Santos et al., 2015 [19]	15 months	Male	After neurosurgical intervention	Yes	*E. coli, K. pneumoniae*	IV, IVT, and IT	Meropenem	Recovered
Santos et al., 2015 [19]	11 months	Male	After neurosurgical intervention	Yes	*A. baumannii*	IV, IVT, and IT	Meropenem, Amikacin	Recovered
Tekgunduz et al., 2015 [20]	2 months	Male	After neurosurgical intervention	Yes	*A. baumannii*	IV, IVT, and IT	Gentamicin, Sulbactam	Moderate disability
Mahabeer et al., 2018 [21]	1 month	Male	Healthcare-associated infection	Yes	*A. baumannii*	IV, IVT, and IT	Gentamicin	Recovered
Hiremath et al., 2018 [22]	17 years	Female	After neurosurgical intervention	Yes	*A. baumannii*	IVT and IT (11 days)	IV meropenem and teicoplanin	recovered
Abad-Restrepo et al., 2018 [23]	11 years	Female	After neurosurgical intervention	Yes	*P. aeruginosa*	IV, IVT, and IT (42 days)	Vancomycin	Recovered
AlZailaie et al., 2018 [24]	5 years	Female	After neurosurgical intervention	Yes	*A. baumannii*	IV, IVT, and IT (49 days)	--	Recovered
Al Yazidi et al., 2018 [25]	<1 month	Male	After neurosurgical intervention	Yes	*Enterobacter cloacae*	IV, IVT, and IT (9 days)	Meropenem, Ciprofloxacin	Died
Hussain et al., 2021 [26]	1 month	Female	After neurosurgical intervention	Yes	*E.coli, K. pneumoniae*	IV, IVT, and IT (7 days)	IV meropenem, vancomycin	Recovered
Hussain et al., 2021 26]	<1 month	Male	After neurosurgical intervention	Yes	*A. baumannii*	IV, IVT, and IT (7 days)	IV meropenem, vancomycin	Recovered
Hussain et al., 2021 [26]	< 1 month	Male	Healthcare-associated infection	Yes	*A. baumannii*	IV, IVT, and IT (5 days)	IV meropenem, vancomycin	Recovered
Hussain et al., 2021 [26]	<1 month	Female	After neurosurgical intervention	Yes	*A. baumannii*	IV, IVT, and IT (8 days)	IV cefotaxime, Meropenem, amikacin, colistin	Recovered
Hussain et al., 2021 [26]	<1 month	Male	Healthcare-associated infection	Yes	*A. baumannii*	IV, IVT, and IT (7 days)	IV cefotaxime, Meropenem, amikacin	Moderate disability
Hussain et al., 2021 [26]	<1 month	Male	Healthcare-associated infection	Yes	*A. baumannii*	IV, IVT, and IT (3 days)	IV cefotaxime, Meropenem, amikacin, vancomycin	Died
Hussain et al., 2021 [26]	<1 month	Male	After neurosurgical intervention	Yes	*K pneumoniae, A. baumannii*	IV, IVT, and IT (8 days)	IV meropenem, Ceftazidime, amikacin	Recovered

EVD: External ventricular drain, MDR: Multidrug resistant, NR: Not reported, IVT: Intraventricular therapy, and IT: Intrathecal.

## Data Availability

All data generated or analyzed during this study are included in this article. Further enquiries can be directed to the corresponding author.

## Supplementary Materials

The following supporting information can be downloaded at: https://www.mdpi.com/article/10.3390/tropicalmed7030041/s1. Table S1: Summary of the main findings of included studies.
