# Supplementary material for: Intrathecal/Intraventricular Colistin for Antibiotic-Resistant Bacterial CNS Infections in Pediatric Population: A Systematic Review
Source: Trop Med Infect Dis. 2022 Mar 3;7(3):41. doi: 10.3390/tropicalmed7030041 (PMC8954222; doi:10.3390/tropicalmed7030041)
Supplement: Supplementary file 1 [file tropicalmed-07-00041-s001.zip › tropicalmed-1587290-supplementary.pdf]

Table S1. Summary of the main findings of included studies.

Reference	Age	Gender	Route of infection	EVD	CSF culture	Colistin (duration)	Other antibiotics	Outcome
Kaplan & Patrick, 1990[6]	4 years	NR	CSF leak after trauma	Yes	*A. baumannii*	IV, IVT, and IT (20 days)	--	Recovered
Fernandez-Viladrich et al., 1999[7]	16 years	Male	After neurosurgical intervention	Yes	*A. baumannii*	IVT and IT (20 days)	Meropenem, Tobramycin, Sulbactam	Severe disability
Ng et al., 2006[8]	4 years	Male	After neurosurgical intervention	No	*A. baumannii*	IV, IVT, and IT (24 days)	Amikacin	Severe disability
Yagmur & Esen, 2006[9]	16 years	Male	After neurosurgical intervention	Yes	*P. aeruginosa*	IVT and IT (21 days)	IV amikacin	Recovered
Dalgic et al., 2009[10]	2 months	Female	After neurosurgical intervention	Yes	*A. baumannii*	IV, IVT, and IT (27 days)	--	Recovered
Dalgic et al., 2009[10]	2 months	Female	After neurosurgical intervention	Yes	*K. pneumoniae*	IV, IVT, and IT (14 days)	Ciprofloxacin	Recovered
Özdemir et al., 2010[11]	3 years	Female	After neurosurgical intervention	Yes	*A. baumannii*	IV, IVT, and IT (35 days)	Meropenem, amikacin, ampicillin	Recovered
Cascio et al., 2010[12]	5 years	Male	After neurosurgical intervention	Yes	*Enterobacter cloacae*	IV, IVT, and IT (14 days)	Teicoplanin, Rifampin, cefazidime	Moderate disability
Saleem et al., 2011[13]	5 months	Male	After neurosurgical intervention	Yes	*A. baumannii*	IV, IVT, and IT (24 days)	--	Died
Saleem et al., 2011[13]	9 months	Male	After neurosurgical intervention	No	*A. baumannii*	IV, IVT, and IT (6 days)	--	Recovered
Saleem et al., 2011[13]	3 months	Female	After neurosurgical intervention	Yes	*A. baumannii*	IV, IVT, and IT (11 days)	--	Recovered
Saleem et al., 2011[13]	9 years	Female	After neurosurgical intervention	Yes	*A. baumannii*	IV, IVT, and IT (21 days)	--	Recovered
Wang et al., 2012[14]	15 years	Male	After neurosurgical intervention	Yes	*A. baumannii*	IV, IVT, and IT (24 days)	Meropenem	Recovered
Karaiskos et al., 2013[4]	18 years	Female	After neurosurgical intervention	Yes	*A. baumannii*	IV, IVT, and IT (17 days)	Carbapenem, Sulbactam.	Recovered
Bargiacchi et al., 2014[15]	18 years	Male	After neurosurgical intervention	Yes	*P. aeruginosa*	IV, IVT, and IT (18 days)	Ciprofloxacin	Recovered
Tekgündüz et al., 2015[16]	<1 month	Male	After neurosurgical intervention	Yes	*A. baumannii*	IV, IVT, and IT (9 days)	Vancomycin	Recovered

Santos et al., 2015[17]	15 months	Male	After neurosurgical intervention	Yes	*E. coli, K. pneumoniae*	IV, IVT, and IT	Meropenem	Recovered
Santos et al., 2015[17]	11 months	Male	After neurosurgical intervention	Yes	*A. baumannii*	IV, IVT, and IT	Meropenem, Amikacin	Recovered
Tekgunduz et al., 2015[18]	2 months	Male	After neurosurgical intervention	Yes	*A. baumannii*	IV, IVT, and IT	Gentamicin, Sulbactam	Moderate disability
Mahabeer et al., 2018[19]	1 month	Male	Healthcare-associated infection	Yes	*A. baumannii*	IV, IVT, and IT	Gentamicin	Recovered
Hiremath et al., 2018[20]	17 years	Female	After neurosurgical intervention	Yes	*A. baumannii*	IVT and IT (11 days)	IV meropenem and teicoplanin	recovered
Abad-Restrepo et al., 2018[21]	11 years	Female	After neurosurgical intervention	Yes	*P. aeruginosa*	IV, IVT, and IT (42 days)	Vancomycin	Recovered
AlZailaie et al., 2018[22]	5 years	Female	After neurosurgical intervention	Yes	*A. baumannii*	IV, IVT, and IT (49 days)	--	Recovered
Al Yazidi et al., 2018[23]	< 1 month	Male	After neurosurgical intervention	Yes	*Enterobacter cloacae*	IV, IVT, and IT (9 days)	Meropenem, Ciprofloxacin	Died
Hussain at al 2021 [24]	1 month	Female	After neurosurgical intervention	Yes	*E.coli, K pneumoniae*	IV, IVT, and IT (7 days)	IV meropenem, vancomycin	Recovered
Hussain at al 2021 [24]	< 1 month	Male	After neurosurgical intervention	Yes	*A. baumannii*	IV, IVT, and IT (7 days)	IV meropenem, vancomycin	Recovered
Hussain at al 2021 [24]	< 1 month	Male	Healthcare-associated infection	Yes	*A. baumannii*	IV, IVT, and IT (5 days)	IV meropenem, vancomycin	Recovered
Hussain at al 2021 [24]	< 1 month	Female	After neurosurgical intervention	Yes	*A. baumannii*	IV, IVT, and IT (8 days)	IV cefotaxime, Meropenem, amikacin, colistin	Recovered
Hussain at al 2021 [24]	< 1 month	Male	Healthcare-associated infection	Yes	*A. baumannii*	IV, IVT, and IT (7 days)	IV cefotaxime, Meropenem, amikacin	Moderate disability
Hussain at al 2021 [24]	< 1 month	Male	Healthcare-associated infection	Yes	*A. baumannii*	IV, IVT, and IT (3 days)	IV cefotaxime, Meropenem, amikacin, vancomycin	Died
Hussain at al 2021 [24]	< 1 month	Male	After neurosurgical intervention	Yes	*K pneumoniae, A. baumannii*	IV, IVT, and IT (8 days)	IV meropenem, Ceftazidime, amikacin	Recovered

EVD: External ventricular drain MDR: Multidrug resistant NR: Not reported

IVT: Intraventricular therapy ITH: Intrathecal,
